# Pulmonary hepatoid adenocarcinoma: report of a case

**DOI:** 10.1186/s40792-016-0129-6

**Published:** 2016-01-08

**Authors:** Yamato Motooka, Kentaro Yoshimoto, Takashi Semba, Koei Ikeda, Takeshi Mori, Yumi Honda, Ken-ichi Iyama, Makoto Suzuki

**Affiliations:** Department of Thoracic Surgery, Faculty of Life Sciences, Kumamoto University, 1-1-1 Honjo, Chuo-ku, Kumamoto, 860-8556 Japan; Department of Surgical Pathology, Kumamoto University Hospital, 1-1-1 Honjo, Chuo-ku, Kumamoto, 860-8556 Japan

**Keywords:** Hepatoid adenocarcinoma, Alpha-fetoprotein (AFP), Lung neoplasms

## Abstract

Hepatoid adenocarcinoma (HAC) is a rare neoplasm with aberrant hepatocellular differentiation. HAC occurs in extrahepatic organs such as the gastrointestinal tract, testes, ovaries, and lungs and frequently produces alpha-fetoprotein. A 69-year-old patient was diagnosed clinically with T2aN0M0, stage IB, non-small cell lung carcinoma. Because the tumor showed tight adhesion to the chest wall, we performed left upper lobectomy, combined resection of the 3rd and 4th ribs, and lymph node dissection. Pathological examination confirmed the diagnosis of HAC of the lung (pathological T2aN0M0, stage IB), and four courses of cisplatin and gemcitabine were administered as adjuvant chemotherapy. Genetic analysis of the epidermal growth factor receptor showed wild type. Preoperative serum alpha-fetoprotein level, a useful marker of disease progression, was elevated to 4497 ng/ml, decreasing within the normal range by about 3 months postoperatively. The patient remains alive without recurrence as of 51 months after surgery.

## Background

Hepatoid adenocarcinoma (HAC) is a rare neoplasm showing aberrant hepatocellular differentiation. HAC occurs in extrahepatic organs such as the gastrointestinal tract, testes, ovaries, and lungs and frequently produces AFP. HAC in any extrahepatic organs is reportedly associated with poor prognosis. We describe herein a case of pulmonary HAC treated by complete resection and adjuvant chemotherapy.

## Case presentation

**Fig. 1:**
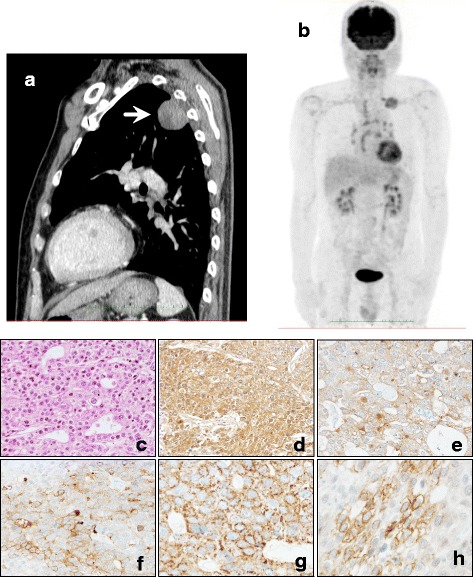
**a** Chest CT. A mass shadow is present in the left upper lung field (*arrow*). **b** Fluorodeoxyglucose-positron emission tomography shows abnormal uptake in the left lung tumor, bilateral hilar lymph nodes, and left supraclavicular lymph nodes. **c** Neoplastic cells show hepatocellular-like differentiation and a partial pseudo-biliary tract pattern (hematoxylin and eosin staining). **d** Immunohistochemical staining for alpha-fetoprotein shows focal positivity. **e** Immunohistochemical staining for carcinoembryonic antigen shows focal positivity. **f** Immunohistochemical staining for glypican 3 shows focal positivity. **g** Immunohistochemical staining for hepatocyte-specific antigen shows focal positivity. **h** Immunohistochemical staining for protein induced by vitamin K shows focal positivity

In March 2011, chest X-ray detected abnormal shadows in the left lung of a 69-year-old man with a 40-pack-year history of smoking. In 2006, he underwent surgical resection of early tongue cancer, which was identified as squamous cell carcinoma. Chest computed tomography (CT) revealed a mass lesion in the left segments 1+2, with a diameter of 4.3 cm (Fig. 1a). The tumor was subpleural and in close contact with the parietal pleura, and a right lower paratracheal lymph node (#4R) was found to be swollen (diameter, 1.0 cm). Fluorodeoxyglucose-positron emission tomography (FDG-PET) showed abnormal uptakes in the left lung tumor (maximum standard uptake value = 5.7), bilateral hilar lymph nodes, and left supraclavicular lymph nodes (Fig. 1b). CT-guided needle biopsy confirmed poorly differentiated squamous cell carcinoma in the left lung tumor. The #4R lymph node showed no malignant findings on endobronchial ultrasonography-transbronchial needle aspiration biopsy (EBUS-TBNA), so we diagnosed clinical T2aN0M0, stage IB, non-small cell lung carcinoma. Surgery performed in June 2011 revealed tumor adhering tightly to the chest wall, so we performed left upper lobectomy, combined resection of the 3rd and 4th ribs, and lymph node dissection.

The resected specimens showed hepatocellular features such as a pseudo-biliary tract pattern (Fig. 1c). Immunohistochemical staining showed tumor cells with focal positivity for AFP (Fig. 1d), carcinoembryonic antigen (Fig. 1e), glypican 3 (Fig. 1f), hepatocyte-specific antigen (Fig. 1g), protein induced by vitamin K absence or antagonist (Fig. 1h), and CD56. In contrast, stains for cytokeratin (CK)7, CK20, thyroid transcription factor 1, surfactant apoprotein, CK5/6, p63, involucrin, chromogranin A, and synaptophysin all yielded negative results. The tumor showed no vessel invasion, lymphatic involvement, or lymph node metastasis. Although the tumor conglutinated to the chest wall, no invasion of the thoracic wall was evident on histopathological examination. Pathological examination confirmed a diagnosis of HAC of the lung (pathological T2aN0M0, stage IB, according to the 7th edition TNM classification). Examination for mutations in the gene for epidermal growth factor receptor (*EGFR*) was conducted using polymerase chain reaction assay, showing wild-type *EGFR*. Given the diagnosis of HAC, we examined preoperative serum AFP levels in stored blood samples. AFP was found to be elevated to 4497 ng/ml at 1 month preoperatively, decreasing within the normal range postoperatively. The patient received four courses of cisplatin and gemcitabine as adjuvant chemotherapy and remains alive without recurrence as of 51 months after surgery.

## Discussion

Some cases of AFP-producing lung carcinoma were reported in the 1980s. Ishikura et al. first reviewed seven cases of AFP-producing lung carcinoma in 1990 and diagnosed five of the seven cases with HAC [1]. Since that time, 18 cases of HAC of the lung have been reported [1–10], clarifying the clinicopathological and immunohistochemical features. According to those reports, HAC is associated with large tumor, AFP production, poor prognosis, a predilection for older patients, regional lymphadenopathies, and distant metastases [2, 6]. Some studies have described long-term survival of 2–7 years [6–9]. These cases were all in the early pathological stage, with two cases in stage IIB, one in stage IIA, and one in stage IB. Our case was likewise in stage IB and has so far survived 51 months postoperatively. From the above findings, curative resection at an early stage could contribute to long-term survival.

HAC is an extremely aggressive tumor with poor prognosis, and effective chemotherapies have yet to be established. On the other hand, survival for 9 years and for 37 months has been reported in patients with clinical stage IV HAC in the lung [10]. Those patients received multimodal therapy comprising surgery/tumor debulking and chemoradiotherapy, but no description of the specific regimen was provided. The results, however, suggest that intensive treatment can improve patient survival. We were conducting a clinical trial of cisplatin (CDDP) and gemcitabine (GEM) as adjuvant chemotherapy for patients with completely resected pathological stage IB-IIIA non-small cell lung carcinoma (UMIN000005256) at the time we encountered the present patient and enrolled him in the trial. A previous study suggested a survival advantage of adjuvant chemotherapy for stage IB patients who had tumors ≥4 cm in diameter [11], which was why we designed this study cohort to include stage IB tumors.

The possibility of pulmonary metastasis from hepatocellular carcinoma was excluded for the lung tumor in this case, because no liver tumors were apparent on preoperative contrast-enhanced CT, FDG-PET, or postoperative ultrasonography. Metastasis from hepatocellular carcinoma of the stomach or other organs was also ruled out. HAC of the lung was therefore diagnosed.

In this case, serum AFP levels decreased from 4497 ng/ml at 44 days preoperatively to 125.5 ng/ml at 25 days after surgery and within normal range by 82 days after surgery. From that time, serum AFP level has remained within normal limits and no recurrence has been identified. Serum AFP level could offer a useful marker of disease progression for HAC.

In our case, EBUS-TBNA was useful for accurate staging. As the absence of lymph node metastases was confirmed by EBUS-TBNA, the patient was able to undergo surgery while the tumor was still in the early stage.

## Conclusions

In conclusion, we encountered a case of HAC in the lung, representing a rare neoplasm with aberrant hepatocellular differentiation. The patient underwent complete resection and adjuvant chemotherapy with CDDP+GEM and as of the time of writing has survived without recurrence for 51 months postoperatively. Although more cases are required, our findings suggest that complete resection and multimodal therapy can improve the prognosis for patients with HAC in the lungs.

## Consent

Written informed consent was obtained from the patient for publication of this case report and the accompanying images. A copy of the written consent is available for review by the editor in chief of this journal.

## Abbreviations

AFP: alpha-fetoprotein
CK: cytokeratin
CT: computed tomography
EBUS-TBNA: endobronchial ultrasonography-transbronchial needle aspiration biopsy
EGFR: epidermal growth factor receptor
FDG-PET: fluorodeoxyglucose-positron emission tomography
HAC: hepatoid adenocarcinoma
